# The Rice Aspartyl-tRNA Synthetase YLC3 Regulates Amino Acid Homeostasis and Chloroplast Development Under Low Temperature

**DOI:** 10.3389/fpls.2022.847364

**Published:** 2022-03-04

**Authors:** Hongjia Liu, Xue Gong, Hui Deng, Jinjuan Tan, Yanqing Sun, Fang Wang, Wenjuan Wu, Zhongjing Zhou, Rumeng Xu, Haiyan He, Clive Lo

**Affiliations:** ^1^State Key Laboratory for Managing Biotic and Chemical Threats to the Quality and Safety of Agro-Products, Institute of Virology and Biotechnology, Zhejiang Academy of Agricultural Sciences, Hangzhou, China; ^2^College of Life Sciences, Fujian Agriculture and Forestry University, Fuzhou, China; ^3^Institute of Crop Science and Institute of Bioinformatics, Zhejiang University, Hangzhou, China; ^4^Shanghai Key Laboratory of Plant Molecular Sciences, College of Life Sciences, Shanghai Normal University, Shanghai, China; ^5^School of Biological Sciences, The University of Hong Kong, Hong Kong, Hong Kong SAR, China

**Keywords:** rice, aspartyl-tRNA synthetase, chloroplast, amino acid metabolism, eiF2α

## Abstract

Aminoacyl tRNA synthetases primarily function to attach specific amino acids to the corresponding tRNAs during protein translation. However, their roles in regulating plant growth and development still remain elusive. Here we reported a rice thermo-sensitive mutant *yellow leaf chlorosis3* (*ylc3*) with reduced chlorophyll content, altered thylakoid structure, and substantially elevated levels of free aspartate, asparagine and glutamine in leaves under low temperature condition. Map-based cloning identified that *YLC3* encodes an aspartyl-tRNA synthetase which is localized in cytosol and mitochondria. In addition, quantitative proteomics analysis revealed that both nuclear and chloroplast-encoded thylakoid proteins were significantly down-regulated in the mutant. On the other hand, proteins involved in amino acid metabolism and the process of protein synthesis were up-regulated in *ylc3*, particularly for key enzymes that convert aspartate to asparagine. Moreover, uncharged tRNA-Asp accumulation and phosphorylation of the translation initiation factor eIF2α was detected in the mutant, suggesting that YLC3 regulates the homeostasis of amino acid metabolism and chloroplast thylakoid development through modulation of processes during protein synthesis.

## Introduction

Rice is one of the most important staple crops in the world, feeding more than half of Asia’s population. Photosynthesis efficiency is a major determinant of crop productivities. Chloroplasts are the location not only for photosynthesis, but also for biosynthesis of many important metabolites. Originated from endosymbiotic cyanobacteria approximately 1 billion years ago, chloroplasts are semi-autonomous organelles capable of independent transcription and translation (Dyall et al., 2004; Jarvis and López-Juez, 2013). Over a long period of symbiotic evolution, the majority of chloroplast genes had been transferred to the nuclear genome, and their encoded proteins are translated in cytosol and then translocated to chloroplasts (Woodson and Chory, 2008). Currently there are approximately 3,000 chloroplast proteins, among which only about 100 proteins are encoded by the chloroplast genome (Abdallah et al., 2000; Richardson et al., 2017). Signaling regulatory mechanisms exist between nuclear genes and chloroplast genes. There are numerous nuclear genes regulating the processes of transcription, post-transcriptional modification, and translation in chloroplasts (Fernández and Strand, 2008). On the other hand, chloroplasts are regulating nuclear gene expression through tetrapyrrole signals and its own redox status (Fernández and Strand, 2008). Meanwhile, mitochondria, as the cellular energy factories which generate a large amount of ATP, are important for chloroplast biogenesis and development. In the rice mutant *wp3*, mitochondrial functional deficiencies also resulted in inhibition of chloroplast development (Li et al., 2018). Hence, normal chloroplast development requires coordinated regulation by nucleus, chloroplasts, and mitochondria.

Homeostasis of amino acid metabolism is pivotal to cellular growth and development (Bröer and Bröer, 2017). During amino acid deficiencies, mammalian-specific protein kinases are activated and they phosphorylate the translation initiation factor eiF2α (Hinnebusch, 2005; Wek et al., 2006). Consequently, translation of most proteins is inhibited to reduce energy consumption. At the same time, the expression of genes encoding enzymes for amino acid biosynthesis is enhanced to ensure cellular survival by transcription factor GCN4 (General Control Non-derepressible-4) during nutritional deficiencies (Natarajan et al., 2001; Dever and Hinnebusch, 2005; Li and Lam, 2008). In yeast, the GCN2 (General Control Non-derepressible-2) kinase-mediated eIF2α phosphorylation is an important adaptation strategy for metabolic and physiological changes resulting from nutritional deficiencies (Dever et al., 1992). Interaction between GCN1 and GCN2 is a necessary condition for the activation of GCN2 (Marton et al., 1993; Sattlegger and Hinnebusch, 2005). The Arabidopsis AtGCN2 could complement the yeast *gcn2* mutant phenotypes, implicating functional conservation (Zhang et al., 2003). In addition, chemically-induced amino acid starvation, UV light, wounding, chilling, and hormone treatments in Arabidopsis could all cause AtGCN2-dependent eIF2α phosphorylation which requires AtGCN1 and AtGCN2 interactions (Lageix et al., 2008; Zhang et al., 2008; Li et al., 2013; Wang et al., 2017). Moreover, Arabidopsis *atgcn1-1* and *atgcn1-2* mutants are cold-stress sensitive with retarded chloroplast development (Zhang et al., 2017). A recent study in Arabidopsis revealed that change in reactive oxygen species (ROS) levels in chloroplasts could also initiate AtGCN2-mediated eIF2α phosphorylation (Lokdarshi et al., 2020). These results indicated that eIF2a phosphorylation is a cold-stress tolerance mechanism in plants through inhibition of protein translation.

Aminoacyl-tRNA synthetases (AARSs) are one of the key enzymes involved in protein synthesis by linking amino acids with their specific tRNA (Ibba and Soll, 2000; O’Donoghue and Luthey-Schulten, 2003). Plant proteins are synthesized in cytosol, mitochondria and chloroplasts which all have a complete set of AARSs. In plants, AARSs are all nuclear-encoded, translated in cytosol, and then translocated to cytosol, mitochondria, or chloroplasts. There are 45 AARSs identified in Arabidopsis and some of them are translocated to different organelles to ensure normal protein synthesis (Duchêne et al., 2005). It can be perceived that sharing of AARSs between organelles is imperative for regulation of translation since the activities of AARSs and tRNA charged state are the major control points for prokaryotic and eukaryotic translational systems (Duchêne et al., 2005).

Gene cloning and functional characterizations of AARS mutants in tobacco, Arabidopsis, and rice revealed the involvement of some mitochondrial- and chloroplast-targeted AARSs chloroplast development and their mutations resulted in chlorotic or albino phenotypes (Kim et al., 2005; Liu et al., 2007; Wang et al., 2016; Zhang et al., 2017; Fang et al., 2020). The glutamyl-tRNA synthetase encoded by rice *Os10g0369000* is localized in cytosol and mitochondria, showing anther-specific expression and participating in anther formation and development (Yang et al., 2018). In Arabidopsis, 21 out of the 45 known AARSs are necessary for endosperm formation and embryogenesis (Berg et al., 2005). For example, cysteinyl-tRNA synthetase is specifically expressed in central cells of female gametes, determining the fate of the adjacent accessory cells (Kägi et al., 2010). Meanwhile, the Arabidopsis aspartyl-tRNA synthetase (IBI1, Impaired in BABA-induced disease Immunity1) is a beta-aminobutyric acid-inducible receptor protein for broad-spectrum disease resistance. IBI1 is mainly localized in endoplasmic reticulum and cytoplasm. Its deficiency led to accumulation of uncharged tRNAs, which in turn promoted GCN2-dependent phosphorylation of eIF2α, thereby inhibiting plant growth and development (Luna et al., 2014). In addition, IBI1 can be localized in nucleus, interacting with the transcription factors VOZ1/2 (Vascular Plant One Zinc Finger1/2) and regulating the expression of cell-wall defense and abiotic stress-responsive genes through the abscisic acid signaling pathway (Schwarzenbacher et al., 2020). Plant AARSs are mainly affecting amino acid homeostasis, protein synthesis and abiotic stress (Luna et al., 2014; Yang et al., 2018; Schwarzenbacher et al., 2020). However, it remains largely elusive regarding the protein localization and expression pattern of most of the other AARSs and their roles in plant growth and development, particularly for those in monocot species like rice.

In the present study, we reported the function of a cytosol- and mitochondria-localized aspartyl-tRNA synthetase in rice. YLC3 functional deficiency disrupted amino acid homeostasis, initiated eIF2α phosphorylation, inhibited translation, and influenced chloroplast development under low temperature condition.

## Results

### Phenotypic Characterization of the *ylc3* Mutant

**FIGURE 1:**
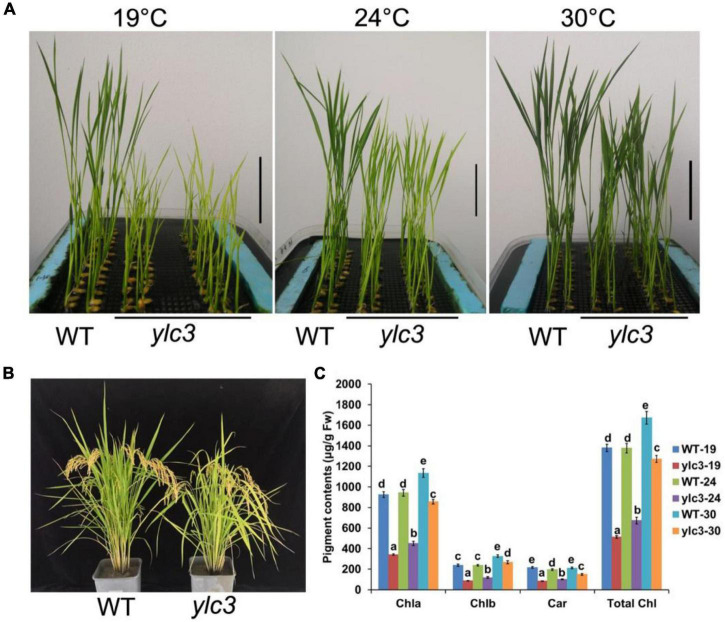
Phenotypic analyses of *ylc3* mutant. **(A)** Phenotypes of the WT and *ylc3* seedlings (10-day-old) grown at different temperatures. Bar = 5 cm. **(B)** Heading stage. **(C)** Photosynthetic pigments of WT and *ylc3* (10-day-old) grown at different temperatures (°C). Error bars represent SD (*n* = 5). Bars with different letters indicate significant differences at *P* < 0.05, ANOVA.

**FIGURE 2:**
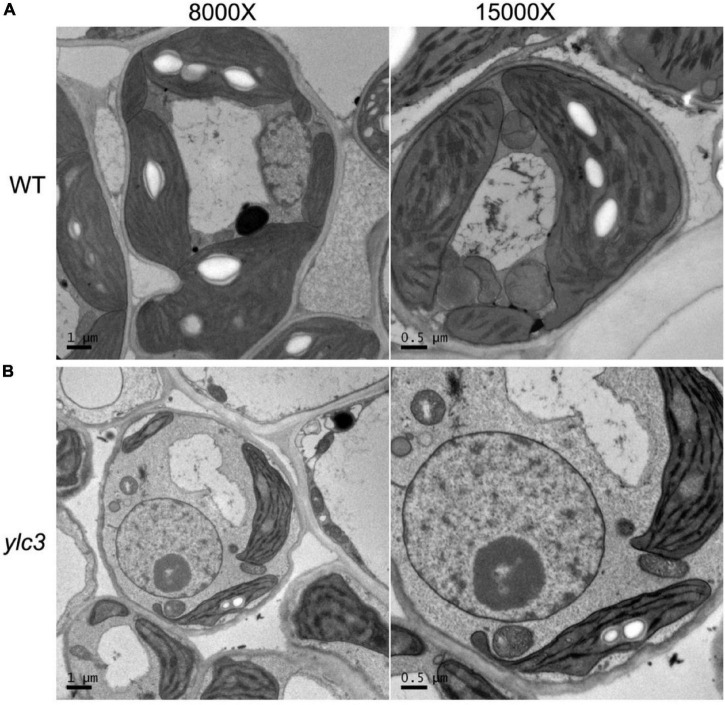
Impaired thylakoid development in *ylc3* mutant. **(A)** WT chloroplasts showed well-developed thylakoids. **(B)** *ylc3* chloroplasts showed reduced number of thylakoid grana lamella.

From a population of ethyl methanesulfonate (EMS)-induced rice mutants, a yellow leaf mutant designated as *ylc3* (*yellow leaf chlorosis3*) was identified. At 19°C, 2-week-old *ylc3* mutant plants showed yellow leaves containing only 30% photosynthetic pigments compared to wild-type plants. The photosynthetic pigments increased to 50 and 80% of wild-type levels at 24 and 30°C, respectively. In addition, chlorophyll a content was inhibited more pronouncedly than chlorophyll b in the mutant. These data indicated that *ylc3* is a thermo-sensitive mutant (Figure 1). In light growth chambers, plant growth was affected by different temperatures. Under field conditions, *ylc3* plants were slightly shorter than wild-type plants while heading period and seeding rate were both normal (Figure 1). Transmission electronic microscopy analysis revealed the impaired development of chloroplasts in *ylc3* growing at 19°C with substantially reduced number of thylakoid grana lamella (Figure 2).

### Map-Based Cloning of the *YLC3* Gene

**FIGURE 3:**
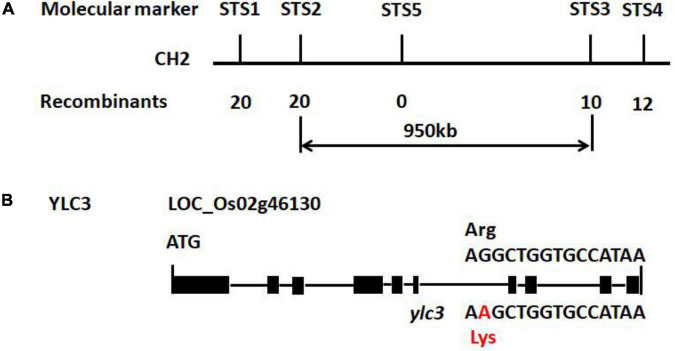
Preliminary gene mapping and *ylc3* mutation. **(A)** InDel markers (STS1-5) used for preliminary mapping are indicated. Numbers of recombinants and F_2_ mutants (n) are shown. **(B)** Gene structure of *YLC3* and the mutation site. The “A” in red indicates a one base-pair substitution in *ylc3*. Black rectangles represent exons.

To study the genetics of the yellow leaf phenotype, *ylc3* was crossed separately with Nipponbare or Kasalath wild-type plants. Under low temperature condition, the F_2_ population of the *ylc3* × Nipponbare cross showed a segregation ratio of 95:330 for yellow leaf vs. green leaf(1:3 ratio, χ^2^ = 2.38 < χ^2^_0_._05_ = 3.84, *P* > 0.05); 129 of 409 plants showed yellow leaf phenotype in the F_2_ generation of *ylc3* × Kasalath (1:3 ratio, χ^2^ = 0.30 < χ^2^_0_._05_ = 3.84, *P* > 0.05). The above results indicate that the yellow leaf phenotype is conferred by a single recessive nuclear gene. F_2_ population from the *ylc3* × Kasalath cross was then used for genetic mapping. In the preliminary mapping, 94 F_2_ plants with yellow leaf phenotype were analyzed with 120 sequence-tagged site (STS) markers which are evenly distributed on 12 rice chromosomes. *YLC3* was initially mapped within a 950-kb region between the molecular markers STS2 and STS3 on chromosome 2. At the same time, F_2_ population from the *ylc3* and Nipponbare cross was subject to whole-genome sequencing-based MutMap method to map *YLC3* (Abe et al., 2012; Lü et al., 2015). The mutation was identified to be a single-base substitution (G → A) in the aspartyl-tRNA synthetase-encoding gene located on chromosome 2 (*LOC_Os02g46130*), resulting in a single amino acid replacement (Arginine → Lysine) near the C-terminal region of YLC3 (Figure 3).

**FIGURE 4:**
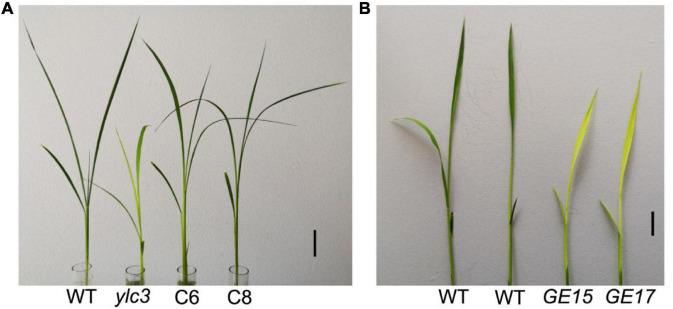
Complementation and *YLC3* gene editing analysis. **(A)** The *ylc3* mutation was complemented by *YLC3* promoter expression of a full-length YLC3 coding sequence. Bar = 3 cm. **(B)** *YLC3* gene-editing transgenic seedlings showed the yellow leaf phenotype at low temperature. Bar = 1 cm.

To validate that *LOC_Os02g46130* was the affected gene, we constructed the binary vector Pro:YLC3-NOS for *ylc3* transformation. A total of 20 independent transformants were obtained with wild-type phenotypes. Meanwhile, a gene-editing vector was constructed for Nipponbare transformation. A total of 22 positive transformants were obtained, 9 of which were homozygous for the G → A substitution and they all showed yellow leaf phenotype at low temperature condition. Phenotyping and genotyping analyses revealed the yellow leaf phenotype in the T_1_ plants growing at lower temperatures. The above results established that the yellow leaf phenotype was conferred by the *ylc3* mutation in *LOC_Os02g46130* (Figure 4).

### Sequence and Phylogenetic Analyses

**FIGURE 5:**
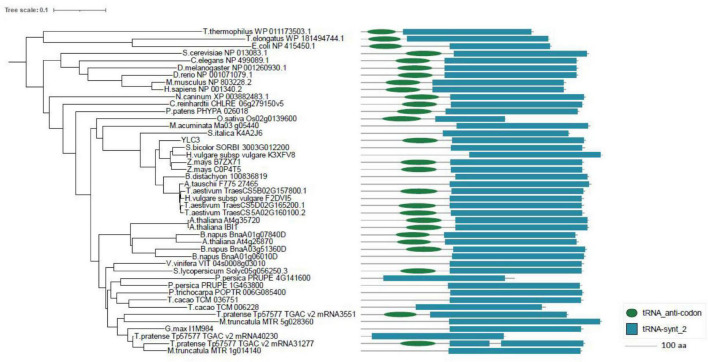
Phylogenetic analysis and motif alignment. A phylogenetic tree was constructed with aligned full-length sequences of homologs of YLC3. Amino acid sequences from regions 101 to 188 and 223 to 544 in YLC3 were used for motif alignment by MEGA.

Aminoacyl-tRNA synthetases are classified into two categories: eukaryotic-specific (clade I) and prokaryotic-originated (clade II). Sequence analysis of YLC3 revealed the presence of an N-terminal coiled coil functional domain and a C-terminal tRNA synthetase class II functional domain, hence YLC3 belongs to a clade II AARS. Clustering analysis further illustrated that YLC3 is very conserved among different species with highly homologous functional domain and motif. Among angiosperms, YLC3 shares higher homology with proteins from monocotyledons such as sorghum, barley and corn, with slightly lower homology with proteins from dicot species such as Arabidopsis, *Brassica napus* and soybean (Figure 5). Taken together, YLC3 is a highly conserved clade II aspartyl-tRNA synthetase.

### Expression and Subcellular Localization Analyses

**FIGURE 6:**
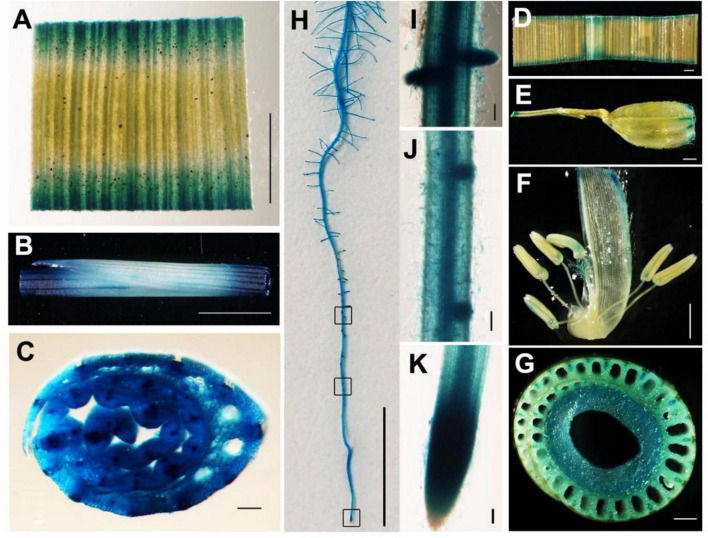
Tissue expression pattern of *YLC3*. **(A–C,H–K)** GUS staining of 7-day-old ProYLC3:GUS transgenic seedlings. **(A)** Leaf. **(B)** Stem. **(C)** Cross section of stem. **(H)** Primary root. **(I)** Lateral root. **(J)** Lateral root primordium. **(K)** Root tip. **(D–G)** GUS staining of the heading stage of ProYLC3:GUS transgenic plants. **(D)** Leaf. **(E)** Glume. **(F)** Anthers and pistils. **(G)** Cross section of stem. Scale bars are 100 μm in **(C,D,E,I–K)**, 1 mm in **(A,B,F,G)**, and 1 cm in **(H)**.

To examine the spatial expression of *YLC3*, a promoter: GUS binary vector was constructed and transformed into Nipponbare rice. GUS staining was performed in roots, stems, leaves, glumes, anthers, and pistils from the positive transformants. Results indicated that *YLC3* was expressed in all tissues examined. GUS staining was stronger in roots, stems, and leaves but weaker in anthers and pistils (Figure 6). Hence, *YLC3* is considered a constitutively expressed gene.

**FIGURE 7:**
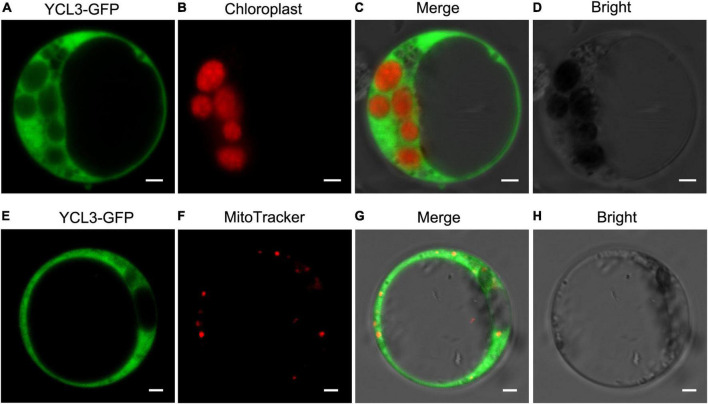
Subcellular localization of YLC3-sGFP. **(A–D)** Transient expression of YLC3:sGFP in rice protoplasts. **(A)** GFP. **(B)** Chlorophyll autofluorescence. **(C)** Merged images of **(A,B)**. **(D)** Bright field. **(E–H)** Transient expression of YLC3:sGFP and MitoTracker staining of transfected protoplasts. **(E)** GFP. **(F)** Mitochondrial fluorescence staining by MitoTracker. **(G)** Merged images of **(E,F)**. **(H)** Bright field.

Aminoacyl-tRNA synthetases are mainly participating in protein synthesis and there is a complete set of AARSs in cytosol, mitochondria, and chloroplasts. To examine the subcellular localization of YLC3, a 35S promoter-driven YLC3:sGFP fusion expression vector was constructed. Transient expression of YLC3:sGFP in rice protoplasts revealed green fluorescent signals mainly in cytosol (Figure 7). After staining the transfected protoplasts with a mitochondrial specific dye (MitoTracker, Invitrogen, Carlsbad, CA, United States), some YLC3:sGFP green fluorescent signals were found to overlap with the red mitochondrial signals (Figure 7). These observations indicated that YLC3 is mainly localized in cytosol and mitochondria, but not in chloroplasts.

### Analysis of Free Amino Acids in *ylc3* Mutant

**FIGURE 8:**
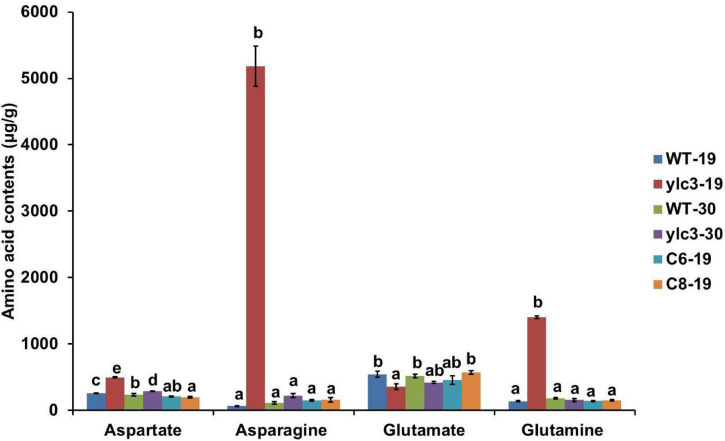
Free amino acid analysis of 10-day-old WT and ylc3 seedlings grown at different temperatures. Error bars represent SD (*n* = 3). Bars with different letters indicate significant differences at *P* < 0.05, ANOVA.

*YLC3* encodes an aspartyl-tRNA synthetase which catalyzes the reaction between aspartate and its specific tRNA. If aspartyl-tRNA synthetase is functionally deficient, it may result in the accumulation of free aspartate and uncharged tRNAs. Wild type plants, *ylc3* mutant plants and complementation lines were kept at low temperature (19°C) and high temperature (30°C) in light growth chambers and free amino acid contents in leaves were determined at 2-leaf stage. Under low temperature condition, *ylc3* mutant leaves showed 92% increase in aspartate, 10-fold increase in glutamine, and 78-fold increase in asparagine, when compared to wild-type plants and complementation lines (Figure 8). Under high temperature condition, contents of aspartate, glutamine, and asparagine were restored to normal levels in the *ylc3* mutant leaves (Figure 8). The above results demonstrated the changes in free amino acid contents in *ylc3* leaves, especially asparagine, under low temperature condition.

### Quantitative Proteomics Analyses

Since AARSs are playing pivotal roles in protein synthesis while *ylc3* mutant showed severe inhibition of chlorophyll production and chloroplast development under low temperature condition, quantitative proteomics analyses were performed with *ylc3* and wild-type seedlings growing at 19°C. Using the high-throughput tandem mass tag method, a total of 9,212 proteins comprising 9143 nuclear-encoded proteins, 51 chloroplast-encoded proteins, and 18 mitochondrial-encoded proteins were detected (Supplementary Table 1). Among the mitochondrial-encoded proteins, 11 proteins were up-regulated in *ylc3* seedlings while the others were unaffected. All the 25 chloroplast-encoded proteins in different thylakoid protein complexes such as Photosystem I, Photosystem II, cytochrome *b_6_f* complex, and NADH dehydrogenase complex were down-regulated in the mutant (Table 1).

**TABLE 1:** Chloroplast-encoded thylakoid protein with significant fold changes in *ylc3*.

Accession	Description	Pathway analysis	Ratio(M:W)	Adjust_*p*_value
1073625666	PetD (chloroplast) [*Oryza sativa*]	Electron transport system	0.246	1.91E-07
1073625664	PsbH (chloroplast) [*Oryza sativa*]	Photosystem II	0.282	9.97E-08
1073625661	PsbB (chloroplast) [*Oryza sativa*]	Photosystem II	0.334	3.18E-08
1073625622	PsbC (chloroplast) [*Oryza sativa*]	Photosystem II	0.377	8.04E-08
1073625653	PsbE (chloroplast) [*Oryza sativa*]	Photosystem II	0.445	2.09E-07
1073625621	PsbD (chloroplast) [*Oryza sativa*]	Photosystem II	0.499	3.34E-07
1073625651	PsbL (chloroplast) [*Oryza sativa*]	Photosystem II	0.624	3.92E-06
1073625616	PsbA (chloroplast) [*Oryza sativa*]	Photosystem II	0.573	5.52E-07
1073625649	PetA (chloroplast) [*Oryza sativa*]	Electron transport system	0.515	2.02E-07
1073625699	NdhA (chloroplast) [*Oryza sativa*]	Electron transport system	0.591	5.90E-07
1073625639	NdhJ (chloroplast) [*Oryza sativa*]	Electron transport system	0.626	5.54E-06
1073625640	NdhK (chloroplast) [*Oryza sativa*]	Electron transport system	0.678	4.16E-06
1073625691	NdhF (chloroplast) [*Oryza sativa*]	Electron transport system	0.681	6.36E-06
1073625684	NdhB (chloroplast) [*Oryza sativa*]	Electron transport system	0.727	0.000157
1073625698	NdhI (chloroplast) [*Oryza sativa*]	Electron transport system	0.729	1.62E-05
1073625700	NdhH (chloroplast) [*Oryza sativa*]	Electron transport system	0.740	2.92E-05
1073625695	PsaC (chloroplast) [*Oryza sativa*]	Photosystem I	0.349	1.99E-07
1073625636	PsaA (chloroplast) [*Oryza sativa*]	Photosystem I	0.399	1.70E-07
1073625635	PsaB (chloroplast) [*Oryza sativa*]	Photosystem I	0.499	1.76E-07
1073625631	AtpH (chloroplast) [*Oryza sativa*]	ATPase	0.383	4.25E-06
1073625630	AtpI (chloroplast) [*Oryza sativa*]	ATPase	0.393	1.39E-07
1073625632	AtpF (chloroplast) [*Oryza sativa*]	ATPase	0.452	2.61E-07
1073625642	AtpE (chloroplast) [*Oryza sativa*]	ATPase	0.479	1.41E-07
1073625643	AtpB (chloroplast) [*Oryza sativa*]	ATPase	0.471	1.32E-07
1073625633	AtpA (chloroplast) [*Oryza sativa*]	ATPase	0.522	3.76E-07

The KEGG analysis of 4,979 nuclear-encoded proteins revealed 1,384 differentially expressed proteins (>2-fold changes) with 154 proteins down-regulated and 1,230 proteins up-regulated (173 of them were up-regulated by more than threefold) in the *ylc3* mutant (Supplementary Table 2). The down-regulated proteins are enriched in photosynthesis-related proteins including 13 photosynthetic antenna proteins, 20 photosystem-related proteins, 14 photosynthetic carbon fixation proteins, and 14 carbohydrate metabolism-related proteins (Table 2). Most of the nuclear-encoded and chloroplast-encoded thylakoid membranes were apparently down-regulated, which was probably the major cause for the reduced chlorophyll content, impaired thylakoid development, and yellow seedling phenotype in the *ylc3* mutant.

**TABLE 2:** Nuclear-encoded thylakoid protein with significant fold changes in *ylc3.*

Accession	Description	Gene_name	Pathway Analysis	Ratio (M:W)	Adjust_*p*_value
LOC_Os01g52240.1	Similar to type I chlorophyll a/b-binding protein b (fragment)	Lhcb1.1	Photosystem II	0.231	3.49E-08
LOC_Os04g38410.1	Similar to chlorophyll a/b-binding protein CP24, photosystem II (fragment)	CP24	Photosystem II	0.244	3.49E-08
LOC_Os02g37060.1	Similar to photosystem II 5 kD protein		Photosystem II	0.258	3.51E-08
LOC_Os08g01380.1	Ferredoxin I, chloroplast precursor (anti-disease protein 1)	Fd1	Photosystem II	0.2667	4.44E-07
LOC_Os01g64960.1	22-kDa photosystem II protein, photoprotection	PSBS1	Photosystem II	0.269	3.03E-08
LOC_Os05g22730.1	Similar to one helix protein		Photosystem II	0.274	8.19E-08
LOC_Os03g39610.1	Similar to photosystem II type II chlorophyll a/b binding protein (fragment)	LHCB	Photosystem II	0.277	2.82E-08
LOC_Os07g37240.1	Similar to chlorophyll a/b-binding protein CP29 precursor	CP29	Photosystem II	0.288	3.37E-08
LOC_Os07g37550.1	Similar to type III chlorophyll a/b-binding protein (fragment)		Photosystem II	0.297	3.89E-08
LOC_Os07g36080.1	Similar to oxygen-evolving enhancer protein 3-2, chloroplast precursor		Photosystem II	0.335	5.60E-08
LOC_Os08g10020.1	Similar to photosystem II 10 kDa polypeptide (fragment)	OsPsbR3	Photosystem II	0.335	6.37E-08
LOC_Os01g31690.1	Similar to photosystem II oxygen-evolving complex protein 1 (fragment)	PsbO	Photosystem II	0.368	1.48E-07
LOC_Os09g17740.1	Similar to chlorophyll a-b binding protein, chloroplast precursor (LHCII type I CAB) (LHCP)	CAB1R	Photosystem II	0.388	7.93E-08
LOC_Os02g10390.1	Chlorophyll a/b-binding protein type III (fragment)		Photosystem II	0.402	9.30E-08
LOC_Os07g04840.1	Similar to 23 kDa polypeptide of photosystem II	PsbP	Photosystem II	0.46	2.85E-07
LOC_Os03g19380.1	Similar to CP12 (fragment)	OsCP12	Photosystem II	0.507	1.61E-06
LOC_Os03g21560.1	Similar to photosystem II 11 kD protein		Photosystem II	0.508	5.22E-07
LOC_Os08g44680.1	Similar to Photosystem I reaction center subunit II, chloroplast precursor (photosystem I 20 kDa subunit) (PSI-D)	PsaD	Photosystem II	0.509	1.79E-07
LOC_Os09g26810.1	Similar to type II chlorophyll a/b binding protein from photosystem I precursor	Lhca6	Photosystem II	0.52	3.26E-07
LOC_Os03g17174.1	Similar to kinase binding protein (fragment)	PsbP	Photosystem II	0.529	2.34E-07
LOC_Os06g01210.1	Plastocyanin, chloroplast precursor	OsPC	Electron transport system	0.282	5.66E-07
LOC_Os01g01340.1	Light-regulated protein, regulation of light-dependent attachment of LEAF-TYPE FERREDOXIN-NADP + OXIDOREDUCTASE (LFNR) to the thylakoid membrane	LIR1	Electron transport system	0.325	5.35E-07
LOC_Os08g45190.1	Similar to PGR5	OsPGR5	Electron transport system	0.371	1.19E-07
LOC_Os02g01340.2	Leaf-type ferredoxin-NADP+-oxidoreductase, regulation of electron partitioning in the chloroplast	OsLFNR1	Electron transport system	0.508	2.40E-07
LOC_Os04g33830.1	Photosystem I PsaO domain containing protein	PsaO	Photosystem I	0.216	7.02E-08
LOC_Os09g30340.1	Similar to photosystem I reaction center subunit V	PsaG	Photosystem I	0.277	6.85E-08
LOC_Os06g21590.1	Similar to light-harvesting complex I (fragment)		Photosystem I	0.296	1.30E-07
LOC_Os12g23200.1	Similar to photosystem I reaction center subunit XI, chloroplast precursor (PSI- L) (PSI subunit V)	PsaL	Photosystem I	0.317	1.27E-07
LOC_Os12g08770.1	Similar to photosystem I reaction center subunit N, chloroplast precursor (PSI- N)	PsaN	Photosystem I	0.369	3.08E-06
LOC_Os08g33820.1	Similar to LHC I type IV chlorophyll binding protein (fragment)	cab	Photosystem I	0.398	1.19E-07
LOC_Os05g48630.2	Photosystem I reaction center subunit VI, chloroplast precursor (PSI- H) (light-harvesting complex I 11 kDa protein) (GOS5 protein)	PSAH	Photosystem I	0.409	1.99E-07
LOC_Os07g25430.1	Photosystem I reaction center subunit IV, chloroplast precursor (PSI- E) (photosystem I 10.8 kDa polypeptide)	PsaE	Photosystem I	0.426	2.11E-07
LOC_Os02g52650.1	Similar to Lhca5 protein		Photosystem I	0.427	1.92E-07
LOC_Os07g05480.1	Photosystem I protein-like protein	PsaK	Photosystem I	0.429	1.18E-06
LOC_Os03g56670.2	Similar to photosystem-1 F subunit	OsPS1-F	Photosystem I	0.44	1.01E-07
LOC_Os03g52130.1	Photosystem I reaction center subunit N family protein		Photosystem I	0.517	5.00E-05

**FIGURE 9:**
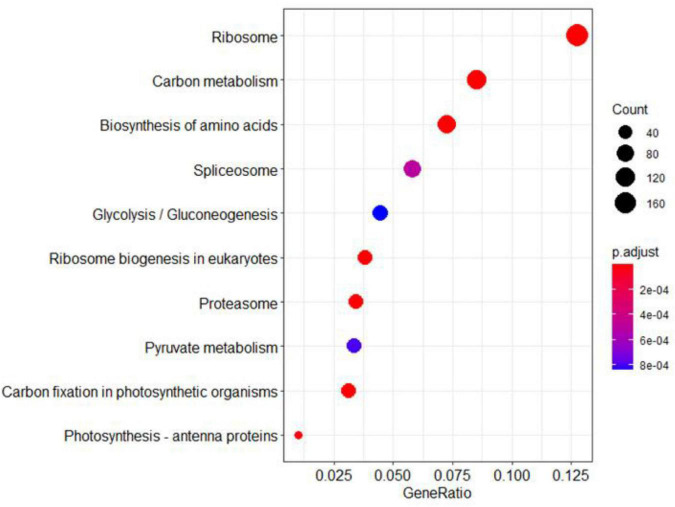
KEGG pathway analysis. Differentially regulated proteins were used to perform KEGG analysis on clusterProfiler. Significant differences between *ylc3* and WT were detected for proteins related to photosynthesis. Pathways including carbon metabolism, pyruvate metabolism, carbon fixation in photosynthetic organisms and photosynthesis-antenna proteins were enriched (corrected *P*-value < 0.05).

The KEEG analysis also identified 166 ribosomal proteins, 92 carbon metabolism-related proteins, 88 amino acid biosynthesis proteins, and 41 pyruvate metabolism-related proteins (Figure 9). Most of these proteins were significantly up-regulated in the *ylc3* mutant. For the aspartate metabolic pathways, asparagine synthetase, glutamine synthetase, and aspartate aminotransferase were all up-regulated while glutamate synthetase did not show any significant changes (Table 3). The above results suggested that ribosomal protein translation efficiencies were increased and amino acid biosynthesis was enhanced, especially for asparagine and glutamine, in the *ylc3* mutant under low temperature condition.

**TABLE 3:** Expression levels of aspartate metabolic pathway enzymes in *ylc3.*

Accession	Description	Gene name	Ratio (M:W)	Adjust_*p*_value
LOC_Os02g50240.1	Glutamine synthetase	OsGS1;1	1.418	9.96025E-06
LOC_Os02g50240.2	Glutamine synthetase	OsGS1;1	1.383	0.000125334
LOC_Os03g50490.1	Glutamine synthetase	OsGS1;3	1.991	9.37E-07
LOC_Os03g12290.1	Glutamine synthetase	OsGS1;2	1.503	5.85E-06
LOC_Os01g48960.1	Glutamate synthase	OsNADH-GOGAT1	0.998	0.955012272
LOC_Os05g48200.1	Glutamate synthase	OsNADH-GOGAT2	1.161	0.001536878
LOC_Os02g14110.1	Aminotransferase	Aspartate aminotransferase	1.594	1.78E-06
LOC_Os01g55540.1	Aminotransferase	Aspartate aminotransferase	2.363	1.19E-07
LOC_Os06g35540.1	Aminotransferase	Aspartate aminotransferase	2.318	1.77E-07
LOC_Os03g18130.1	Asparagine synthetase	Aspartate synthetase	2.704	5.93E-07

### Uncharged tRNA-Asp Accumulation and Immunoblot Analysis of eif2α Phosphorylation

In Arabidopsis, aspartyl-tRNA synthetase deficiency resulted in accumulation of aspartate and uncharged tRNA, which in turn interacted with AtGCN2 and activated its kinase activities. The activated AtGCN2 then phosphorylated eIF2a, reduced protein translation efficiencies, and inhibited plant growth and development (Luna et al., 2014). Two aspartyl-tRNA genes (trnD-GUC, Id: 29141347; trnD-GTC, Id: 3950710) in rice were retrieved from the NCBI database. To check uncharged tRNA-Asp levels, northern-blot analysis was performed. Our results demonstrated that tRNA-trnD-GUC was obviously increased in *ylc3* seedlings under low temperature. However, we failed to detect the transcription of trnD-GTC in rice seedlings.

**FIGURE 10:**
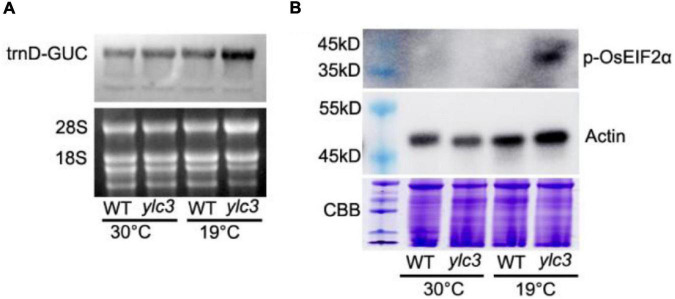
Uncharged tRNA-Asp accumulation and phosphorylation of OsEIF2α at different growth temperatures. **(A)** Uncharged tRNA-Asp levels in WT and *ylc3* seedlings were analyzed by northern blot. The ethidium bromide-stained gel is shown as a loading reference. **(B)** Phosphorylation levels of OsEIF2α in WT and *ylc3* seedlings were monitored by immunoblot analysis. Upper and lower panels represent immunoblot results using antibodies against phosphorylated eIF2α and actin, respectively. Coomassie Brilliant Blue (CBB) staining is shown as a loading reference. Protein size marker is indicated.

We speculated that the eif2α phosphorylation level might be increased in the *ylc3* mutant under low temperature condition. Accordingly, AtEIF2α, AtGCN1, and AtGCN2 protein sequences were searched against the NCBI rice database to retrieve the homologous proteins in rice as OsEIF2α (LOC_Os03g18510), OsGCN1 (LOC_Os03g51140) and OsGCN2 (LOC_Os04g41530), respectively. These three proteins were found to be up-regulated by onefold from the above quantitative proteomics analysis. Since the phosphorylation sites are identical between OsEIF2α and AtEIF2α (as revealed by sequence alignment), eif2α-specific phosphorylation antibody (Luna et al., 2014; Wang et al., 2017) could be utilized for immunoblot detection in rice plants. We found that eif2α was apparently phosphorylated in the *ylc3* mutant growing under low temperature condition when compared to the wild-type plants (Figure 10). Hence, under low temperature condition, *ylc3* mutation resulted in eif2α phosphorylation which could inhibit the translation of thylakoid complex proteins, leading to impaired chloroplast development and yellow leaf phenotype.

## Discussion

Plant AARSs with different expression patterns and subcellular locations play key role in protein synthesis (Ibba and Soll, 2000; Duchêne et al., 2005). YLC3, a cytosol- and mitochondrial-localized aspartyl-tRNA synthetase, is required for free amino acid homeostasis in rice under low temperature condition. In addition, translation of thylakoid proteins is likely to be down-regulated by the GCN2-eif2α phosphorylation pathway in the *ylc3* seedlings.

### *YLC3* Encodes an Aspartyl-tRNA Synthetase

*YLC3* is a constitutively expressed gene with stronger expression in roots, stem and leaves but weaker expression in anthers and pistils (Figure 6). YLC3 contains the typical aspartyl-tRNA catalytic domain and a coiled coil functional domain. Phylogenetic analysis illustrated that YLC3 is highly conserved with reported aspartyl-tRNA synthetases including Arabidopsis IBI1 (Luna et al., 2014). Furthermore, free amino acid analyses revealed that aspartate level was increased in the *ylc3* mutant. Collectively, the enhanced aspartate level together with the protein functional domain and phylogenetic analyses strongly suggested that YLC3 is an aspartyl-tRNA synthetase.

The rice *ylc3* mutant is a thermo-sensitive chlorotic mutant and gene mapping identified a single amino acid substitution in the C-terminal region of YLC3. The substitution occurred outside the catalytic and coiled coil domains, indicating that the C-terminus is also critical for the activities of aspartyl-tRNA synthetase. The importance of this substitution was further supported by gene editing approach which generated transgenic rice lines with the same mutation and phenotype. On the other hand, chlorophyll and free amino acid contents are normal in *ylc3* plants under high temperature condition, indicating that the mutant YLC3 protein can be functional. Alternatively, as there are at least 3 aspartyl-tRNA synthetases in rice, it remains to be investigated whether the other isozymes are involved in restoration of chlorophyll and free amino acid contents under high temperature conditions.

There is a lack of an N-terminal signal peptide in YLC3 which is localized in cytosol and mitochondria, but not chloroplasts (Figure 7). Currently, all the known aminoacyl-tRNA synthetases participating in chloroplast development are chloroplast-localized. The Arabidopsis aspartyl-tRNA synthetase IBI1 is localized in endoplasmic reticulum and cytosol. During pathogen attack or low temperature stress, IBI1 is translocated to nucleus, inducing immunity responses in Arabidopsis (Schwarzenbacher et al., 2020). It cannot be excluded that YLC3 is translocated to chloroplasts to participate in chloroplast development under low temperature condition.

### Changes of Amino Acid Homeostasis in *ylc3* Mutant

**FIGURE 11:**
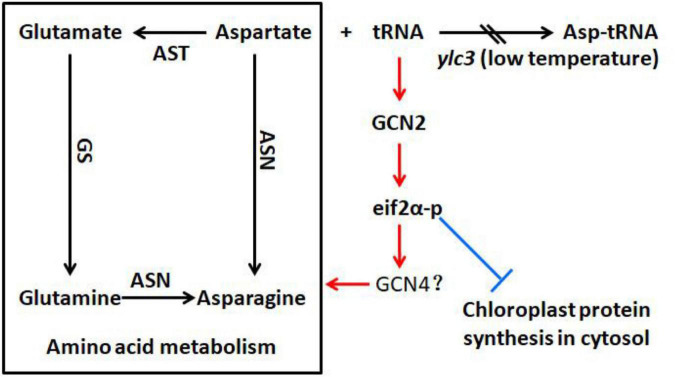
Possible involvement of YLC3 in amino acid homeostasis and chloroplast development. Loss of YLC3 function leads to accumulation of uncharged tRNAs, which promote GCN2-dependent phosphorylation of eIF2α (eIF2α-p), thereby up-regulating amino acid synthesis and inhibiting chloroplast development. Consequently, excess free aspartate is converted to glutamine and asparagine for storage. Positive and negative regulatory effects are indicated in red and blue colors, respectively. ASN, asparagine synthetase; AST, aspartate aminotransferase; GS, glutamine synthetase.

We have measured the 20 free amino acids in leaves of *ylc3* mutant growing under low temperature condition. Surprisingly, asparagine was increased by 78-fold, glutamine was increased by 10-fold while aspartate was only increased by 92% compared to wild-type plants. Meanwhile, Arabidopsis *ibi1* mutant showed 100% increase in aspartate (Luna et al., 2014). Rice *osers1* mutant anthers contain 144% more glutamate, 76% more aspartate, and 168% more histidine (Yang et al., 2018). According to the aspartate metabolic pathway, aspartate aminotransferase converts aspartate to glutamate and oxaloacetate, then glutamine synthetase converts glutamate to glutamine, and finally asparagine synthetase converts glutamine and aspartate to asparagine (Figure 11). Consistently, quantitative proteomics analysis demonstrated the up-regulation of the above enzymes with asparagine synthetase (ASN1) being most elevated. A previous study indicated that asparagine synthesis is mainly dependent on ASN1 (Ohashi et al., 2015; Luo et al., 2018). As the free amino acids were restored to normal levels in the *ylc3* complementation lines, the over-accumulation of glutamine and asparagine under low temperature condition represents a consequence of *ylc3* mutation. Consistently, the mutant showed normal levels of free amino acids under high temperature condition.

Apparently, the *ylc3* mutation resulted in functional deficiency of the encoded aspartyl-tRNA synthetase under low temperature condition. Consequently, aspartate and uncharged tRNA-ASP levels would be elevated (Figure 10A). We speculate that most of the excess aspartate had been converted to glutamine and asparagine which are the major storage form of organic nitrogen, some of them could be stored in xylem and phloem sap (Urquhart and Joy, 1981; Lea et al., 2007; Gaufichon et al., 2010, 2016). On the other hand, the conversion may relieve the inhibitory effects of high concentrations of aspartate (Schultz et al., 1998). Meanwhile, it is unknown whether over-accumulation of asparagine and glutamine may affect chloroplast development. Functional investigation of aspartyl-tRNA synthetases in different plant species may reveal whether there are conserved mechanisms for regulating aspartate metabolism and whether there are relationships between changes in free amino acid levels and chloroplast development.

### Differential Regulation of Protein Abundances in *ylc3* Mutant

During amino acid starvation, the yeast GCN2 kinase inhibits protein translation and activates amino acid biosynthesis (Natarajan et al., 2001; Dever and Hinnebusch, 2005). This mechanism is at least partially conserved in the Arabidopsis homologous protein (Li et al., 2013; Luna et al., 2014). Amino acid starvation or AARS deficiencies could result in accumulation of uncharged tRNA. AtGCN2 interacts with uncharged tRNA and becomes activated, thereby phosphorylating the translation initiation factor AtEIF2α and leading to inhibition of protein translation (Luna et al., 2014). Under low temperature condition, aspartate accumulation in the *ylc3* mutant could lead to uncharged tRNA accumulation (Figure 10A). Hence, OsGCN2 would become activated to phosphorylate eif2α, leading to inhibition of protein translation (Figure 10B). In fact, there was no a GCN4 homolog in Arabidopsis (Halford et al., 2004; Halford, 2006). It is unclear which OsGCN2-regulated proteins are involved in the adaptation to amino acid starvation in rice. Under low temperature condition, the up-regulation of enzymes involved in amino acid biosynthesis and related pathways, such as those for pyruvate metabolism in *ylc3* mutant, is consistent with the transcriptional up-regulation of amino acid biosynthesis in yeast during amino acid starvation (Dever and Hinnebusch, 2005; Luna et al., 2014). Furthermore, up-regulation of aspartate metabolic enzymes could convert some of the excess aspartate to asparagine and glutamine for storage, implicating a precise regulating mechanism during over-accumulation of aspartate. There was no significant down-regulation of chlorophyll biosynthesis enzymes, suggesting that the reduced chlorophyll content in the *ylc3* mutant was not due to their inhibition. On the other hand, thylakoid complex proteins are largely down-regulated in *ylc3* mutant under low temperature condition.

Translation of cytosolic mRNA is regulated at both global and mRNA-specific levels. For example, uncharged tRNAs-ASP accumulated and activated AtGCN2 in the Arabidopsis *ibi1* mutant (Li et al., 2013; Luna et al., 2014). Recently, inhibition of ribosome loading by activated GCN2 kinase was demonstrated for mRNAs functionally involved in mitochondrial ATP synthesis, chloroplast thylakoids, vesicle trafficking, and translation (Lokdarshi et al., 2020). In our study, the uncharged tRNAs-ASP level was increased and the translation initiation factor eIF2α was phosphorylated in *ylc3* seedlings under low temperature condition (Figure 10). Quantitative proteomics data also indicated that cytosolic mRNA translation of thylakoid proteins was suppressed specifically in the mutant. Overall, our results suggested that YLC3 deficiency could promote the GCN2-eif2α phosphorylation and impaired chloroplast development by suppressing cytosolic mRNA translation in rice (Figure 11). Further investigations are necessary in order to fully understand the functions of YLC3 and other aspartyl-tRNA synthetases in rice under different stress conditions.

## Materials and Methods

### Plant Materials and Growth Conditions

The rice (*Oryza sativa* ssp. *japonica* cv. Nipponbare) *ylc3* mutant was isolated from an EMS-induced mutant population. Wild-type and *ylc3* mutant plants were grown in Kimura nutrient solution as described previously (Chen et al., 2013). They were kept in a light growth chamber (Panasonic MLR-352H-PC) with a 12 h-light/12 h-dark cycle at 70% relative humidity. Temperatures were set according to each specific treatment.

### Genetic Analysis and Construction of F_2_ Mapping Populations

The *ylc3* mutant was individually crossed with Nipponbare or Kasalath rice to generate the F_1_ progenies which were self-pollinated to obtain the F_2_ population. Using the F_1_ and F_2_ populations, genetic analysis and preliminary mapping were performed. From the F_2_ population of the *ylc3* × Kasalath cross, 22 mutant plants were selected for preliminary mapping. The F_2_ mutant number was increased to 94 during fine mapping. At the same time, genome sequencing and gene cloning were performed using the F_2_ population from the *ylc3* × Nipponbare cross (Abe et al., 2012).

### TEM Analysis of Chloroplast Structures and GUS Staining of Rice Tissues

Fifteen-day-old wild-type and *ylc3* seedlings kept in a light growth chamber at 19°C were used for chloroplast ultra-structural analysis. Leaves were cut into 2-mm sections and fixed using 2.5% glutaraldehyde in cacodylate buffer, following by secondary fixation in OsO_4_. The fixed tissues were dehydrated by ethanol, embedded in epoxy resin, and sectioned for examination under an Hitachi H7650 TEM electron microscope. GUS staining was performed according to Jefferson et al. (1987). Roots, stems, and leaves from 7-day-old seedlings grown at 26°C in a light growth chamber as well as stems, leaves, and panicles from heading stage of mature plants grown in paddy field were collected. Stems and leaves were sliced into 2-mm sections and placed in GUS staining solution for vacuum infiltration (5–10 times) until the samples were completely submerged. After staining at 37°C for 8 h, the tissues were decolored using 70% ethanol and then observed under a Nikon SMZ1000 stereomicroscope.

### Complementation Analysis and Gene Editing Vector Construction

A complementation construct in the pCambia1300 binary vector harboring the YLC3 3.388-kb upstream sequence, the YLC3 1.846-kb cDNA containing the full coding region, and the NOS-3 terminator was generated and named as pCAMBIA1300-PR-YLC3-NOS. It was transformed into *Agrobacterium tumefaciens* EHA105 which was used to infect *ylc3* calluses for 3 days. Afterwards, the calluses were selected on hygromycin plates, followed by differentiation and tissue regeneration (Lee et al., 1999). For construction of the base editing vector, a 19-bp target-specific oligonucleotide initialized by “G” and a 5-bp adaptor were synthesized, annealed and ligated to a *Bsa*I-digested CBEmax-NGG vector (Wang et al., 2019). The constructed vector was confirmed by Sanger sequencing and used for *Agrobacterium*-mediated transformation of rice calluses. For the construction of YLC3 promoter-Gus vector, the 3.388-kb promoter region of YLC3 was fused with the GUS gene in the modified pCAMBIA1300-GUS vector. All primers used for constructing vectors are listed in Supplementary Table 3.

### Subcellular Localization

The *YLC3* coding sequence without a stop codon and fused in-frame with sGFP was cloned into the pCAMBIA1301-35S-NOS vector and transiently expressed in rice protoplasts. Rice seedlings cultured on MS media for 10 days were digested with cellulases for protoplast preparation as described previously (Zhang et al., 2011). Protoplasts (100 μl) were transfected with the vector (5–10 μg) and dark-incubated at room temperature, following by examination of green fluorescence signals under a Zeiss confocal laser scanning microscope. For mitochondria co-localization, transfected protoplasts were stained using a mitochondria fluorescent dye (Mitotracker, Invitrogen, Carlsbad, CA, United States).

### Phylogenetic Analysis and KEGG Analysis

A phylogenetic tree was constructed using aligned full-length sequences of homologs of YLC3. MEGA (version 10.1.7) (Kumar et al., 2018) and the neighbor-joining methods were used with a p-distance model, pairwise deletion and bootstrap (1,000 replicates). The maximum parsimony method of MEGA also was used to support the neighbor-joining tree using the default parameters. Amino acid sequences from regions 101 to 188 and 223 to 544 in YLC3 were used for motif alignment by MEGA. For GO and KEGG analysis, the differentiated expressed proteins were enriched with rice pathways and GO terms using clusterProfiler (Wu et al., 2021) and org.Osativa.eg.db. (Xu, 2019). The filtering criteria of p value Cutoff 0.05 and qvalueCutoff 0.1 were used.

### Measurements of Free Amino Acid Contents

Free amino acid contents in rice leaves were measured using an Hitachi LA8080 automatic amino acid analyzer. Leaves (0.05 g) were placed in 2-ml centrifuge tubes, followed by addition of 1 ml 4% sulfosalicylic acid and two zirconia beads. Bead-beating was performed for 1 h at 1 time per 2 min until the samples became slurry. The samples were allowed to settle for 1 h, followed by centrifugation at 4°C for 10 min. Supernatant (500 μl) was taken, mixed with 500 μl 0.2 M HCl, and filtered through a 0.22 μl millipore filter. Finally, 20 μl sample was used for free amino acid analysis.

### Quantitative Proteomics Analysis of Rice Seedlings

#### Protein Isolation

Crude proteins from rice seedling tissues were extracted by the modified phenol-methanol method as described (Deng et al., 2007). Extracted proteins were dissolved in lysis buffer [8 M urea, 50 mM triethylammonium bicarbonate (TEAB), pH 8.0] and quantified using a 2-D Quant kit (GE Healthcare, Piscataway, NJ, United States) with bovine serum albumin as a standard.

### Tryptic Digestion, Peptide Labeling and Fractionation, LC-MS/MS Analysis

Tryptic digestion, TMT labeling, peptide fractionation and LC-MS/MS were performed as described (Zhu et al., 2022) unless otherwise stated. After tris(2carboxyethyl)phosphin (TCEP) and dithiothreitol (DTT) treatments, proteins were precipitated and dissolved in 50 mM TEAB buffer. Each sample (25 μg) was mixed with its respective 6-plex TMT reagent and incubated for 1 h at room temperature. Three biological replicates were labeled for each sample group, in which the Nipponbare samples were labeled with TMT reagents 126, 127, and 128, while *ylc3* samples were labeled with TMT reagents 129, 130, and 131, respectively. The labeling reactions were stopped by addition of hydroxylamine, then combined and dried by vacuum. The combined multiplexed TMT-labeled samples were separated on a Waters Acquity BEH C18 1.7 μm 2.1–100 mm column using H class UPLC system (Waters, Milford, MA, United States) at a flow rate of 300 μl/min. A total of 24 fractions were collected, then combined into 12 fractions and vacuum dried for LC-MS/MS analysis.

TMT-labeled samples were analyzed on an Ultimate 3000 nano UHPLC system (Thermo Scientific, Waltham, MA, United States) coupled online to a Q Exactive HF mass spectrometer (Thermo Scientific, Waltham, MA, United States) equipped with a Nanospray Flex Ion Source (Thermo Scientific, Waltham, MA, United States). Samples were separated by a binary buffer system of buffer A (0.1% formic acid) and buffer B (80% acetonitrile plus 0.1% formic acid). Peptides were eluted in a gradient of 5–8% solvent B in 2 min, 8–20% solvent B in 66 min, 20–40% solvent B in 33 min, and 40–90% solvent B in 4 min.

### TMT Data Analysis

Raw data were processed using Proteome Discoverer 2.4.0.305 (Thermo Scientific, Waltham, MA, United States) with the SEQUEST HT search engine searching against a rice proteome database (Rice Genome Annotation Project^1^, version 7.0, total 66,338 entries). Searches were configured with static modifications for the TMT reagents on lysine and N-termini, carbamidomethyl on cysteine, dynamic modifications for oxidation of methionine and acetylation of protein N-termini, precursor mass tolerance of 10 ppm, fragment mass tolerance of 0.02 Da, and trypsin cleavage (max 2 missed cleavages). Searches used a reversed sequence decoy strategy to control peptide false discovery and identifications were validated by Percolator software. Protein groups, peptide groups and PSMs were accepted at a false discovery rate (FDR) < 1%. Normalization was applied for the grand total reporter ion intensity for each channel within the 6-plex experiment. Further downstream analysis of the results was performed in the R scripting and statistical environment, using the limma package from Bioconductor^2^. The basic statistics used for significance analysis is the moderated t-statistics. Significantly expressed proteins were filtered for an average fold-change > 1.3 or <0.77, with *p*-values adjusted for multiple testing correction by FDR (Benjamini–Hochberg).

### RNA Isolation and Northern-Blot Analysis

Total RNAs were extracted from 15-day-old seedlings grown at different temperatures using TRIzol reagent (Invitrogen, Carlsbad, CA, United States). RNA was precipitated by ethanol overnight at −20°C. Northern blot was performed as previously described (Huang et al., 2018). The sequences of biotinylated oligomer probes were listed as follows: trnD-GUC (Id: 29141347), 5′-TTGTAGTTCAATTGGTCAGAGCACC-3′; trnD(GTC) (Id: 3950710) 5′-GAAATAGCTCAGTTGGTTAGAGTG-3′.

### Total Protein Extraction and Immunoblot Analysis

The first leaves were collected from WT and *ylc3* mutant seedlings grown at 19 or 30°C for immunoblot analysis. Total proteins were extracted using extraction buffer (containing 25 mM Tris-HCl [pH 7.5], 10 mM NaCl, 4 mM PMSF, 20 mM MG132, and protease inhibitor cocktail). Extracted proteins were subjected to sodium dodecyl sulfate polyacrylamide gel electrophoresis and immunoblotting. Immunoblotting was performed using a monoclonal antibody of phospho-eIF2α (Ser51) (Catalogue no. 9721, Cell Signalling, Danvers, MA, United States 1/1000 dilution) or a monoclonal antibody of Anti Plant Actin Mouse (Abbkine, A01050). Signals were detected using the Immobilon kit (Catalogue no WBKLS0500, Millipore) under standard conditions.

## Data Availability Statement

The proteomics data have been deposited to the ProteomeXchange Consortium. Link: https://www.iprox.cn/page/project.html?id=IPX0004037000; ProteomeXchange ID:PXD031497. The mass spectrometry proteomics data have been deposited to the ProteomeXchange Consortium (http://proteomecentral.proteomexchange.org) via the iProX partner repository (Ma et al., 2019) with the dataset identifier PXD031497.

## Author Contributions

HL and CL designed the experiments. HL, XG, HD, JT, FW, WW, ZZ, RX, and HH performed the experiments. YS, JT, ZZ, HL, and CL analyzed the data. HL and CL wrote the manuscript. All authors contributed to the article and approved the submitted version.

## Conflict of Interest

The authors declare that the research was conducted in the absence of any commercial or financial relationships that could be construed as a potential conflict of interest.

## Publisher’s Note

All claims expressed in this article are solely those of the authors and do not necessarily represent those of their affiliated organizations, or those of the publisher, the editors and the reviewers. Any product that may be evaluated in this article, or claim that may be made by its manufacturer, is not guaranteed or endorsed by the publisher.

## Footnotes

1
http://rice.plantbiology.msu.edu/index.shtml

2
http://www.bioconductor.org/

## Supplementary Material

Supplementary Figure 1 GUS staining of 7-day-old ProYLC3::GUS transgenic seedlings grown at 19°C. Two independent ProYLC3::GUS transgenic lines (pr-1 and pr-4) was used to gus staining test. Bar = 1 cm.

Supplementary Figure 2 Transcriptional expression of YLC3 was performed by Real-time PCR. Gene-specific primers is list below: YLC3-qpF, 5′-CTCCCTCAGC AAGGAATCAA-3′; YLC3-qpR, 5′-CACCTGAATCTCCACCTGCT-3′; Actin-qpF, 5′-GTGTGACAATGGAACTGGCA-3′; Actin-qpR 5′-CCACGATACTAGGGAAAAC AGC-3′.

Supplementary Figure 3 Subcellular localization analysis in YLC3::GFP transgenic rice. Two independent YLC3::GFP transgenic lines cultured on MS media for 10 days were digested with cellulases for protoplast preparation as described previously (Zhang et al., 2011). Green fluorescence signals was observed and captured by a Zeiss confocal laser scanning microscope. For mitochondria co-localization, protoplasts were stained in a mitochondria fluorescent dye (Mitotracker, Invitrogen, Carlsbad, CA, United States).

Supplementary Table 1 9,212 proteins from quantitative proteomics analysis.

Supplementary Table 2 4,979 proteins from KEGG analysis.

Supplementary Table 3 Vector construction primers and indel primers.
