# Supplementary figures and images for: The Rice Aspartyl-tRNA Synthetase YLC3 Regulates Amino Acid Homeostasis and Chloroplast Development Under Low Temperature
Source: Front Plant Sci. 2022 Mar 4;13:847364. doi: 10.3389/fpls.2022.847364 (PMC9635353; doi:10.3389/fpls.2022.847364)

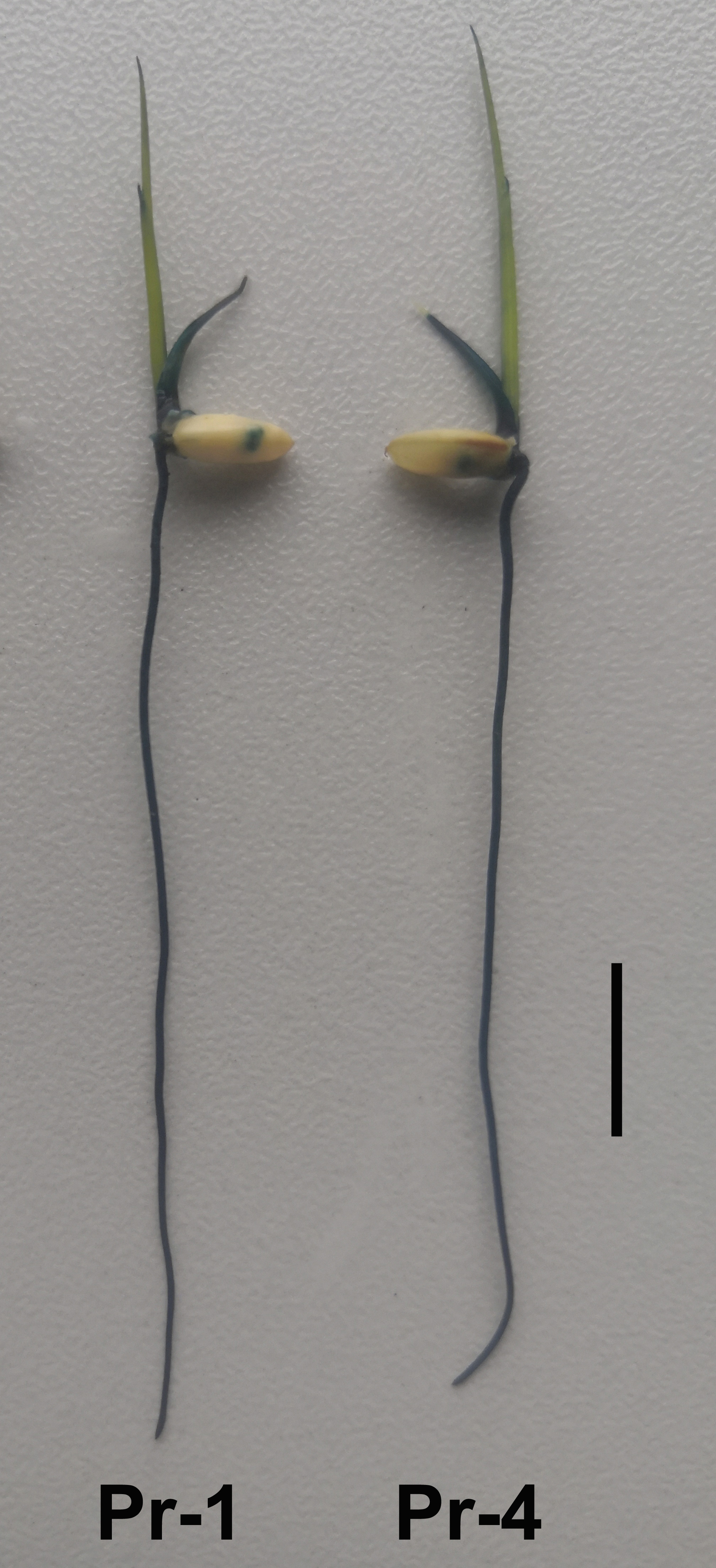

Supplement: Supplementary Figure 1 — GUS staining of 7-day-old ProYLC3::GUS transgenic seedlings grown at 19°C. Two independent ProYLC3::GUS transgenic lines (pr-1 and pr-4) was used to gus staining test. Bar = 1 cm. [file Image_1.JPEG]

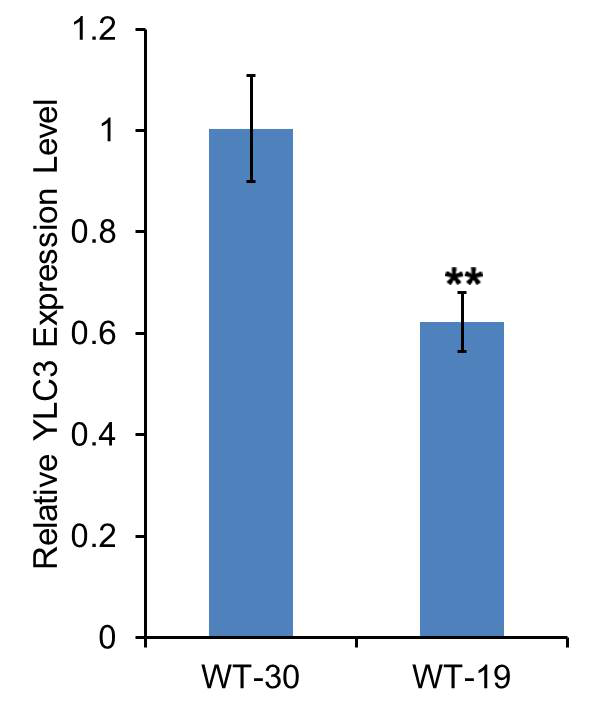

Supplement: Supplementary Figure 2 — Transcriptional expression of YLC3 was performed by Real-time PCR. Gene-specific primers is list below: YLC3-qpF, 5′-CTCCCTCAGC AAGGAATCAA-3′; YLC3-qpR, 5′-CACCTGAATCTCCACCTGCT-3′; Actin-qpF, 5′-GTGTGACAATGGAACTGGCA-3′; Actin-qpR 5′-CCACGATACTAGGGAAAAC AGC-3′. [file Image_2.TIF]

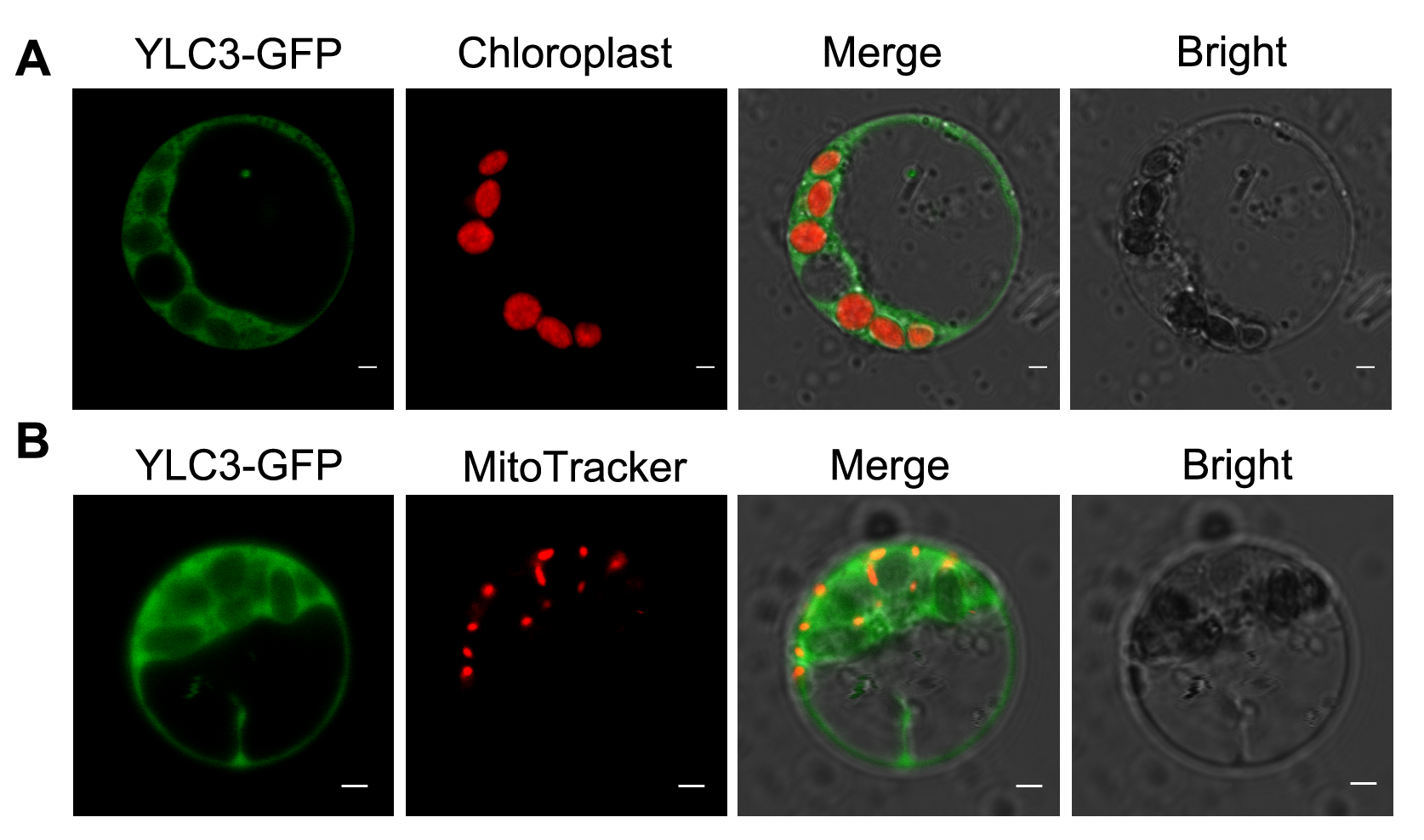

Supplement: Supplementary Figure 3 — Subcellular localization analysis in YLC3::GFP transgenic rice. Two independent YLC3::GFP transgenic lines cultured on MS media for 10 days were digested with cellulases for protoplast preparation as described previously (Zhang et al., 2011). Green fluorescence signals was observed and captured by a Zeiss confocal laser scanning microscope. For mitochondria co-localization, protoplasts were stained in a mitochondria fluorescent dye (Mitotracker, Invitrogen, Carlsbad, CA, United States). [file Image_3.TIF]
