# Supplementary material for: The Rice Aspartyl-tRNA Synthetase YLC3 Regulates Amino Acid Homeostasis and Chloroplast Development Under Low Temperature
Source: Front Plant Sci. 2022 Mar 4;13:847364. doi: 10.3389/fpls.2022.847364 (PMC9635353; doi:10.3389/fpls.2022.847364)
Supplement: Supplementary Table 3 — Vector construction primers and indel primers. [file Table_3.DOCX]

SupTable3. Vector construction primers and indel primers

|  | Primers name | | Primers sequence (5’-3’) |
| --- | --- | --- | --- |
| Gene editing construct primers | YLC3-F | | TGTGTGCAGCCTTTGTGGATCGCGA |
|  | YLC3-R | | AAACTCGCGATCCACAAAGGCTGCA |
| YLC3 promoter-  gus primers  YLC3-  complementation  construct | | YLC3-Pr-567F  YLC3-Pr-3954R  YLC3-106F  YLC3-1951R | CCGGGGATCCTCTAGCGGTTGCAACGAGTAGTGACAC  TACCGAATTCGTCGAGGTAGTTGGCTTGGACTTGGAC  ACGAATTCGAGCTCGGTACCCCAAGTCCAAGCCAACTACC  GCAGGTCGACTCTAGAATGTTGTGGAAAGGAGCAGC |
| Indel primers | STS1-F | | TCGAGCTAATGTGCTTCCAA |
|  | STS1-R | | GCAGAACTCGGTTGCTATCC |
|  | STS2-F | | GCAACGCAGGGAGTATGTTC |
|  | STS2-R | | GCTCACTTCGCTCAACACAA |
|  | STS3-F | | GGCTGACCAATAGCTAGTCTCC |
|  | STS3-R | | CTCCCATTGCCCTATTTTCA |
|  | STS4-F | | GCTTCCCTCACTCTTCCCTT |
|  | STS4-R | | AATAGATCCTGCGTCCCCAG |
|  | STS5-F | | ACCAGCTCTCCATCCATGAA |
|  | STS5-R | | ATGGTCCGGTACATCTGCAA |
